# Microinflammation Factors in the Common Diseases of the Heart and Kidneys

**DOI:** 10.1155/2015/470589

**Published:** 2015-01-14

**Authors:** Danijela Tasic, Sonja Radenkovic, Gordana Kocic, Marina Deljanin Ilic, Aleksandra Ignjatovic

**Affiliations:** ^1^Clinical Centre of Nis, The Clinic of Nephrology, Dr. Zoran Djindjic Boulevard 48, 18000 Nis, Serbia; ^2^The University of Nis, Faculty of Medicine, Dr. Zoran Djindjic Boulevard 81, 18000 Nis, Serbia

## Abstract

*Aim*. To determine levels of interleukin-8 (IL-8) and plasminogen activator inhibitor-1 (PAI-1) in different cardiorenal syndrome (CRS) modalities and to compare findings to some already investigated direct and indirect parameters of inflammation and atherosclerosis. *Materials and Methods*. Testing involved 114 examinees, divided into control and clinical groups suffering from different modalities and were formed according to the basis of a valid classification for CRS. *Results*. C-reactive protein (CRP) was significantly higher in all CRSs in comparison to the control group (*P* < 0.05). PAI-1 in CRSs was statistically higher than in the control group. IL-8 was increased in all CRSs, and especially in CRS-5, where no significance was found. PAI-1 correlated with IL-8 in all CRSs, with significant value in CRS-2 and CRS-5. Correlation for PAI-1 and high-density lipoproteins (HDL) was found in CRS-4, while IL-8 was found to be related to CRP level in all CRSs, with significance only in CRS-1 (*P* < 0.001). *Conclusions*. C-reactive protein, IL-8, and PAI-1 could be useful for clinical differentiation of chronic modalities of CRSs. Inflammation was the most pronounced in CRS-4. Lipid status parameters could be useful for differentiation of CRSs. Furthermore, HDL in chronic primary kidney diseases and triglycerides and total cholesterol in CRS-5 could be valuable.

## 1. Introduction

The cardiorenal syndrome (CRS) is defined as a complex condition involving heart and kidney dysfunction, where acute or chronic disorder of one of these organs induces the acute or chronic disorder of the other organ, resulting in increased cardiovascular morbidity and mortality [1]. The syndrome has been classified into five subtypes (CRS-1 to CRS-5), with different underlying pathophysiological mechanisms, based on the organ primarily involved (heart or kidney) [2]. In spite of different etiology and pathogenesis, common denominators of all CRS modalities are atherosclerosis and inflammation. Numerous investigations have suggested that activity of several inflammation markers in CRSs is amplified in comparison to disorders with single organ involvement [1, 2].

The reactants of the acute and chronic phase of the inflammation process are especially significant microinflammation markers. The acute phase reactants have the role of inhibiting of the inflammation process and are significant in the reestablishment of the homeostasis of the organism. The most significant one in this group is C-reactive protein (CRP), which could indicate direct and indirect tissue damage and the activation of endothelial cells.

IL-8, or CXCL 8 based on the latest nomenclature, is a prototypical chemokine of the CXC subfamily [3]. IL-8 was reported as a particularly important cytokine for estimation of the inflammation process intensity [4]. Although initially IL-8 was recognized primarily as a neutrophil chemoattractant, it was later tested as a potential marker of atherosclerosis and a predictor of occurrence and outcome of acute coronary events. It was also evaluated as a marker of atherosclerosis in patients with high risk (obesity and obesity-related metabolic complications such as diabetes) without direct evidence of developing coronary artery disease (CAD) [5–7].

The plasminogen activator inhibitor-1 (PAI-1) is a member of serine protease inhibitors (SERPINs) family [8]. It is suggested that PAI-1 affects several renal cell signaling mechanisms that are similar to those employed in cardiac muscle tissue [9], with implications for integrity of both organs. PAI-1 is upregulated by inflammatory cytokines and may be considered as a marker of existing inflammatory process, although the underlying mechanism of this is not yet elucidated. Consistently high values of PAI-1 are found in patients with severe sepsis, but also with other acute or chronic inflammatory disorders such as atherosclerosis. Increased plasma levels of PAI-1 are positively correlated with the risk of CAD and myocardial infarction (MI). PAI-1 also seems to be important in pathogenesis of renal diseases. Elevated PAI-1 in plasma was associated with nephritic syndrome and hemolytic uremic syndrome. At present, PAI-1 is considered one of the major markers of endothelial cells' function [10, 11].

Changes in acute and chronic types of cardiorenal syndromes are going through various phases in which inflammation plays an important role. Because of that, markers of inflammation are important in the diagnosis and prognosis of this syndrome.

Based on this, the aim of our investigation was to determine serum levels of IL-8 and PAI-1 in patients with different CRS modalities and to compare our findings to some already investigated direct and indirect parameters of inflammation and atherosclerosis.

## 2. Materials and Methods

One hundred fourteen examinees above the age of 18 of both sexes were involved, divided into the control group, comprising N-35 healthy persons, and clinical groups including 79 patients suffering different modalities of cardiorenal syndrome. In four clinical subgroups with cardiorenal syndrome types 1, 2, 4, and 5 there was almost equal number of patients (CRS-1 N-20, CRS-2 N-22, CRS-4 N-22, and CRS-5 N-13). The smallest number of patients was in subgroup with cardiorenal syndrome type 3 (N-2). Therefore, owing to the low number, this group was excluded from the statistics. The clinical groups were defined based on the internationally recognized classification of cardiorenal syndrome by criteria of the ADQI organization and denoted as CRS-1 to CRS-5. All patients have signed the written informed consent and the study was approved by the Ethic Committee of the Faculty of Medicine in Nis, University of Nis.

The following analyses were done: certain markers of inflammation (C-reactive protein (CRP), interleukin-8 (IL-8), and plasminogen activator inhibitor-1 (PAI-1)), as well as some indirect inflammatory indicators (triglycerides concentrations (TG), total cholesterol (TC), low-density lipoproteins (LDL), and high-density lipoproteins (HDL)) were measured in blood of all persons included in control and clinical groups, in order to evaluate their differential diagnosis capacity in cardiorenal syndrome modalities.

After detailed clinical examination and functional diagnostics, blood samples were drawn from all participants. The full blood samples were centrifuged for 15 minutes on 1000 rpm, followed by separation of 5 mL of serum and frozen to −20°C for determination of C-reactive protein (CRP), interleukin-8 (IL-8), plasminogen activator inhibitor-1 (PAI-1), and concentrations of triglycerides (TG), total cholesterol (TC), low-density lipoproteins (LDL), and high-density lipoproteins (HDL). Standard parameters of lipid status were determined on the automatic analyzer photometrically (ERBA Diagnostics Mannheim XL 600 GMBH, Baden-Wurttemberg, Germany). The serum concentrations of CRP were determined quantitatively, with a nephelometric test (Orion Diagnostica-Turbox). The results were expressed as concentration units (mg/L). Immunoassays of PAI-1 and IL-8 were performed with commercially procured tests, that is, quantitative sandwich enzyme immunoassay technique, and were expressed in ng/mL.

## 3. Statistical Analysis

The statistical analysis was performed with standard data processing programs SPSS 15.0. The values of the tested parameters were presented as an arithmetic average value ± standard deviation (SD) and median. Distributions of the continuous variables were assessed for normality by Shapiro-Wilk test. The difference between independent groups was analysed by an unpaired *t*-test in case of a normal distribution or by Mann-Whitney *U* test if distribution of data was not normal. Spearman (*ρ*) correlation coefficients were used to analyze associations between continuous variables. The rank analysis of variance was performed with the Kruskal Wallis test, owing to inconsistent frequency distribution. The univariate linear regression analysis was performed on all CRS subgroups, in order to test the impact of predictor variables IL-8, CRP, triglycerides concentrations (TG), total cholesterol (TC), low-density lipoproteins (LDL), and high-density lipoproteins (HDL) on the value of the dependent variable of PAI-1. The values of *P* < 0.05 were taken as statistically significant.

## 4. Results

The statistical analysis included a total of 112 patients, divided into control and four clinical groups (CRS-1, CRS-2, CRS-4, and CRS-5). The basic clinical features (sex, age, and BMI) and laboratory parameters of control and CRS groups are given in Tables 1 and 2. Among lipid status parameters, elevated level of TG was found in CRS types 1, 2, and 4, in comparison with control group and CRS-5 group, with statistical significance given in Table 3. Level of TC was significantly increased in CRS type 2 when compared to the value of TC in CRS type 5 (Table 3), while HDL cholesterol was significantly higher in CRS-2 in comparison to CRS-1 (*P* < 0.05), in which HDL had the lowest value (Table 3).

All the investigated parameters, except interleukin-8, showed a difference in the control compared to the clinical group.

Value of CRP was significantly higher in all CRS groups in comparison to the control group (*P* < 0.01, Table 4). In addition, the difference between the subgroups of the clinical group was *P* < 0.05 and was considered less statistically relevant. Level of PAI-1 in CRS groups was also statistically significantly higher than in the control group, with different levels of statistical significance of *P* < 0.01, while the Kruskal Wallis test showed an impact of *P* < 0.05 in all the subgroups (Table 4). Although IL-8 was increased in all CRS groups, and especially in CRS-5, due to extreme values of standard deviation, no statistical significance was found for this parameter (Table 4).

Based on our results, IL-8 could be denoted as only statistically significant predictor of PAI-1 increase (*P* < 0.05) in primary heart disease (CRS-1 and CRS-2) (Tables 5 and 6). The increase per single unit of IL-8 leads to the increase in the value of PAI-1 by 0.02, while in CRS-4 the value of IL-8 was in a statistically significant correlation (*P* < 0.05) with PAI-1 and HDL (*P* < 0.01) (Table 7). An increase per single unit in IL-8 leads to an increase in the value of PAI-1 by 0.004 and by 2.83 in the case of HDL. In CRS-5, statistically significant predictor of PAI-1 value was TG (*P* < 0.05) and TC (*P* < 0.01) (Table 8). An increase per single unit of TG leads to the increase of PAI-1 value by 13.99 and by 9.72 in the case of TC. PAI-1 correlated with IL-8 in all CRS groups, with statistically significant value in CRS-2 and CRS-5 (*P* < 0.05) (Table 9). Correlation for PAI-1 and HDL was found in CRS-4 (*P* < 0.01) (Table 9). IL-8 was found to be related to CRP level in all CRS groups, although with statistical significance only in CRS-1 (*P* < 0.001), as given in Table 10. Strong negative correlation of IL-8 with TC, LDL, and HDL was evident in CRS-1, without statistical significance (Table 10).

## 5. Discussion

Cardiorenal syndrome comprises five groups of cardiac and renal diseases, with different clinical features and pathogenesis [1, 2]. Although heterogeneous, these clinical entities are united by presence of underlying atherosclerosis and inflammation. These processes have several direct biological effects on the cardiovascular and renal systems, leading to both functional and structural and organ damage and dysfunction contributing significantly to the development of CRSs [12]. Today it is considered to be responsible for the development of atherosclerosis and is certainly one of the most important pathogenetic mechanisms in the process of an accelerated atherosclerosis. In care of renal patients accelerated atherosclerosis was noted, which is in the cause of great prevalence of coronary disease, cardiac insufficiency, and diseases of periferal arteries as well as increased mortality [13].

The role of inflammation in the formation of atherosclerosis has been examined in numerous studies related to cardiovascular or renal patients; such an analysis has not been performed in patients who suffer from both cardiac and renal diseases [14, 15]. Some authors have shown that C-reactive protein is an independent predictor of first acute coronary event [16] and “global barometer” of the effectiveness of treatment and modification of risk factors [17]. Levels of serum C-reactive protein did correlate with the presence and severity of coronary, cerebral, and peripheral atherosclerosis in the person with preserved renal function and in patients with different stages of renal failure and dialysis [18]. In this investigation, all patients in CRS groups had an increased serum level of CRP which is in harmony with numerous research findings of other authors. Although CRP was previously treated simply as a marker of inflammation, evidences have been presented that, like the proinflammatory cytokines, CRP exerts detrimental effect on the heart by amplifying the inflammatory response responsible for adverse ventricular remodeling, which could explain especially a significant difference of CRP levels between chronic types of CRS and control group in our study [19, 20].

Literature data suggest that practically every cellular component of the vascular wall could be a potential source of IL-8. Interleukin-8 (IL-8) is a proinflammatory cytokine described as an early marker of acute renal damage. Interleukin-8 has been accepted as a proatherogenic factor and it contributes to the destabilization of the plaque. One study has shown that among 10 cytokines and high-sensitive CRPs (hs-CRPs), IL-8 was the only independent predictor of cardiovascular events during a 7-year follow-up period [21]. Directly and indirectly, through application of IL-8 inhibitors, it was indicated that IL-8 plays a role in pathogenesis of arterial hypertension [22]. All these features were characteristics of patients in CRS involved in this investigation. Besides, our results showed that IL-8, which was already recognized by other authors as an early marker of acute renal injury [23, 24] and predictor of unstable angina pectoris acute myocardial infarction [25], could also serve as a marker for differentiation of CRS-1 from other CRS types, which is also consistent with our examination.

Our results have shown a statistically significant increase of PAI-1 in clinical groups, especially in CRS-4. Some authors suggest that a high level of PAI-1 is related to atherosclerosis and increased risk of plaque rupture [26, 27]. The experimental study suggested presence of profibrotic effect on a muscle myocard, as well as antifibrotic effect in the aging process [28]. An important source of PAI-1 is synthesis and release through sympathetic adrenal axis. Because of that PAI-1 is a key mediator of stress (acute or chronic) induced thrombosis and hypercoagulability [29]. The literature available suggests that the high level of PAI-1 is linked to atherosclerosis and presents an increased risk of plaque rupture [30]. Based on some experimental and clinical findings, an increased level of PAI-1 could be involved in repair processes leading to modulation of local proteolytic potential, thereby governing cell migration, and therefore could be an indicator of ongoing proliferative tissue repair process and progression of atherosclerosis [31]. PAI-1 was recognised as important prognostic factor of acute and important precursor of chronic disease of heart and kidney. Similar studies have not yet been performed in cardiorenal syndrome.

Positive correlation of IL-8 levels and PAI-1 activity means that these are predictors in the pathogenesis of destabilization of chronic heart failure, as it is shown in our results in CRS type 2 [32]. In a state of systemic inflammation that exists in sepsis, finding of elevated values of IL-8 and PAI-1 is one of the mechanisms of insulin resistance and findings of elevated serum glucose level in patients who had no previous diagnosis of diabetes [33]. Our results of IL-8 and PAI-1 showed a significant correlation in CRS-1, CRS-4, and CRS-5 but such studies have not been done in cardiorenal syndrome.

Dyslipidemia is also related to inflammation in chronic renal insufficiency, and total cholesterol (TC) level is one of the indirect indicators of microinflammation [34]. The level of serum triglyceride (TG) is elevated in renal patients, which leads to the endothelial damage and an increased incidence of cardiovascular diseases. In addition, renal insufficiency also has an effect on the accumulation of atherogenic LDL particles, while inflammation reduces the level of HDL, as well as the ability to protect the endothelium from the impact of cytokines and chemokines [35]. Among patients with CRS clinical modalities involved in our study, the most prominent lipid status changes were detected in CRS-5, where TG and TC are statistically the most important, although nonspecific, predictors and indirect markers of underlying inflammatory process.

This single center study hasseveral limitations. Its comparatively small sample size and its cross-sectional nature limit its ability to infer causal relations among variables and might influence the results. The strength of our study is that the patients were well characterized and defined by criteria of the ADQI organisation. This data that reflects “real-world” clinical practice in unselected homogeneous population patients was assigned to ethnicity, gender, and age. A control group has similar qualities in baseline demographic characteristics. Each clinical subgroup, by the number of patients, is less than the control group.

## 6. Conclusion

As a conclusion, among the parameters of inflammations investigated in this research, CRP, IL-8, and PAI-1 were elevated in chronic types of CRS and could be suggested as useful markers for clinical differentiation of chronic modalities of cardiorenal syndrome. Based on our findings, inflammation was the most pronounced in CRS-4. In addition, lipid status parameters, as indirect indicators of inflammation, could be useful for differentiation of CRSs, above all, HDL in chronic primary kidney diseases and triglycerides and total cholesterol in CRS-5. Previous knowledge has shown that numerous factors which are implicated in pathogenesis kidney and heart diseases induce PAI-1 expression. Similar studies have not yet been performed in cardiorenal syndrome.

## Figures and Tables

**Table 1:** Basic clinical features of control and clinical groups.

	Sex M/F	Age mean (range)	BMI mean (kg/m^2^)	Hypertension *N* (%)	Type 2 diabetes mellitus
Control group	17/18	50,14 ± 15,83 (33–67)	25,03 ± 1,81	n/a	n/a

CRS-1 *N* = 20	12/8	68.40 ± 11.09 (54–88)	27,80 ± 2,86	18 (90%)	10 (50.00%)

CRS-2 *N* = 22	12/10	74.95 ± 6.09 (60–84)	28,56 ± 3,31	19 (86.4%)	5 (22.72%)

CRS-4 *N* = 22	16/6	69.27 ± 10.88 (46–86)	26,63 ± 5,80	16 (72.7%)	5 (22.72%)

CRS-5 *N* = 13	10/3	70.15 ± 6.40 (59–80)	24,81 ± 3,09	11 (84.6%)	12 (92.3%)

n/a: not applicable, clinically healthy subjects.

**Table 2:** Basic laboratory parameters in CRS groups.

	CRS-1 *n* = 20	CRS-2 *n* = 22	CRS-4 *n* = 22	CRS-5 *n* = 13	*P* ^*^
Blood glucose (mmol/L)	7,04 ± 2,01	6,43 ± 1,46	6,40 ± 2,54	9,46 ± 5,59	0,226

Creatinine (*μ*mol/L)	456,67 ± 370,21	211,56 ± 154,48	463,34 ± 296,48	320,61 ± 171,04	0,085

Uric acid (mmol/L)	517,15 ± 337,63	413,79 ± 126,28	369,80 ± 114,09	497,66 ± 181,97	0,331

Blood urea (mmol/L)	24,41 ± 17,45	13,95 ± 10,33	21,93 ± 13,63	27,84 ± 14,01	**0,030**

Albumin (g/L)	32,91 ± 4,25	37,42 ± 4,81	35,17 ± 4,93	34,95 ± 7,28	0,078

Data are given as mean value ± SD; ^*^summary *P* was determined with Kruskal-Wallis test. Statistically significant difference is given in bold letters.

**Table 3:** Lipid status in control and CRS groups.

	TG (mmol/L)	TC (mmol/L)	LDL (mmol/L)	HDL (mmol/L)
Control	0.89 ± 0.42 (0.72)	4.08 ± 0.73 (3.87)	2.44 ± 0.62 (2.44)	**1.30 ± 0.29 (1.34)**

CRS-1	2.07 ± 0.84^ac∗∗^ (2.07)	5.03 ± 1.60 (4.48)	3.16 ± 1.33 (2.92)	**1.01 ± 0.29 (0.99)**

CRS-2	2.10 ± 1.00^a∗∗∗c∗^ (1.80)	5.10 ± 1.29^c∗^ (5.02)	2.81 ± 1.31 (2.63)	**1.26 ± **0.30^b∗^ ** (1.30)**

CRS-4	2.05 ± 0.95^a∗∗c∗^ (1.58)	4.90 ± 1.49 (4.67)	2.84 ± 1.23 (2.81)	**1.46 ± 0.91 (1.29)**

CRS-5	1.26 ± 0.52 (1.16)	4.03 ± 0.88 (3.79)	2.21 ± 0.79 (2.50)	**1.12 ± 0.34 (1.07)**

Data are given as mean value SD (median); a: versus control; b: versus CRS-1; c: versus CRS-5; ^*^ *P* < 0.05, ^**^ *P* < 0.01, ^***^ *P* < 0.001.

**Table 4:** Values of CRP, IL-8, and PAI-1 in control and CRS groups.

	CRP (mg/L)	IL-8 (pg/ml)	PAI-1 (ng/ml)
Control	1.27 ± 0.73 (1.10)	35.46 ± 26.05 (26.71)	**3.43 ± 3.62 (2.14)**
CRS-1	82.29 ± 87.23^a∗∗^ (42.80)	110.31 ± 188.38 (38.81)	**8.82 ± **8.11^a∗∗∗^ **(7.29)**
CRS-2	49.01 ± 76.94^a∗∗^ (6.10)	78.58 ± 102.14 (53.28)	**7.72 ± **7.52^a∗^ **(6.60)**
CRS-4	38.83 ± 52.95^a∗∗^ (17.70)	151.12 ± 401.41 (36.16)	**7.78 ± **3.66^a∗∗^ **(9.45)**
CRS-5	72.03 ± 96.94^a∗∗^ (10.70)	405.67 ± 971.56 (61.54)	**7.02 3.43 ± 9.33 (4.88)**

Data are given as mean value ± SD (median).

a: versus control group, ^*^ *P* < 0.05, ^**^ *P* < 0.01, ^***^ *P* < 0.001.

**Table 5:** Results of univariate linear regression analysis for PAI-1 in CRS-1.

	*t*	*P*	OR	95% CI
CRP (mg/L)	1.21	0.2558	0.03	**−0.03–0.09**
IL-8 (pg/ml)	2.11	0.0489^*^	0.02	**0.00–0.04**
TG (mmol/L)	−0.20	0.8443	−0.52	**−6.05–5.02**
TC (mmol/L)	−1.18	0.2586	−1.52	**−4.28–1.25**
LDL (mmol/L)	0.85	0.4174	1.76	**−2.98–6.50**
HDL (mmol/L)	0.18	0.8579	1.71	**−19.29–22.71**

^*^ *P* < 0.05.

**Table 6:** Results of univariate linear regression analysis for PAI-1 in CRS-2.

	*t*	*P*	OR	95% CI
CRP (mg/L)	1.32	0.2081	0.02	**−0.02–0.09**
IL-8 (pg/ml)	2.13	0.0461^*^	0.02	**0.00–0.06**
TG (mmol/L)	−1.12	0.2788	1.81	**−5.86–1.81**
TC (mmol/L)	1.31	0.2083	1.38	**−1.12–4.74**
LDL (mmol/L)	1.11	0.2838	1.47	**−1.52–4.80**
HDL (mmol/L)	−0.11	0.9158	6.40	**−14.32–12.95**

^*^ *P* < 0.05.

**Table 7:** The estimate of the factors relevant to the values of PAI-1 for CRS-4.

	*t*	*P*	OR	95% CI
CRP (mg/L)	−1.36	0.1925	−0.02	**−0.06–0.01**
IL-8 (pg/ml)	2.43	0.0247^*^	0.004	**0.00-0.01**
TG (mmol/L)	0.74	0.4683	0.72	**−1.34–2.77**
TC (mmol/L)	−0.11	0.9139	−0.07	**−1.41–1.27**
LDL (mmol/L)	−0.96	0.3566	−0.82	**−2.70–1.06**
HDL (mmol/L)	3.36	0.0063^**^	2.83	**0.98–4.69**

^*^ *P* < 0.05, ^**^ *P* < 0.01.

**Table 8:** The estimate of the impact of the factors relevant to the value of PAI-1 for CRS-5; the results of the univariate regression analysis.

	*t*	*P*	OR	95% CI
CRP (mg/L)	0.10	0.9253	0.00	−0.08–0.09
IL-8 (pg/ml)	0.62	0.5479	0.002	0.00-0.01
TG (mmol/L)	3.05	0.0226^*^	13.99	2.75–25.23
TC (mmol/L)	5.70	0.0013^**^	9.72	5.54–13.89
LDL (mmol/L)	1.59	0.1736	6.86	−4.26–17.98
HDL (mmol/L)	−0.29	0.7818	−3.25	−30.71–24.21

^*^ *P* < 0.05, ^**^ *P* < 0.01.

**Table 9:** The correlation of PAI-1 with CRP, TG, TC, LDL, and HDL (Spearman coefficient of correlation).

	IL-8 (pg/ml)	CRP (mg/L)	TG (mmol/L)	TC (mmol/L)	LDL (mmol/L)	HDL (mmol/L)
CRS-1	0.33	0.16	−0.17	0.01	0.52	0.32
CRS-2	0.52^*^	0.18	−0.25	0.24	0.17	0.01
CRS-4	0.56^**^	−0.27	0.01	−0.01	−0.24	0.79^**^
CRS-5	0.64^*^	0.22	0.55	0.57	0.14	0.22

^*^ *P* < 0.05, ^**^ *P* < 0.01.

**Table 10:** The correlation of IL-8 with CRP, TG, TC, LDL, and HDL (Spearman coefficient of correlation).

	CRP (mg/L)	TG (mmol/L)	TC (mmol/L)	LDL (mmol/L)	HDL (mmol/L)
CRS-1	0.88^***^	−0.06	−0.49	−0.47	−0.54
CRS-2	0.33	−0.18	0.06	−0.01	−0.12
CRS-4	0.29	0.15	−0.02	−0.02	0.16
CRS-5	0.17	0.14	0.10	−0.29	0.36

^***^ *P* < 0.001.
